# Supplementary material for: Two cytochrome P450 epoxidases mediate juvenile hormone biosynthesis in Drosophila melanogaster
Source: Insect Biochem Mol Biol. Author manuscript; Available in PMC 2026 Jun 22. (PMC13285967; doi:10.1016/j.ibmb.2025.104475)
Supplement: 1 [file NIHMS2187913-supplement-1.pdf]

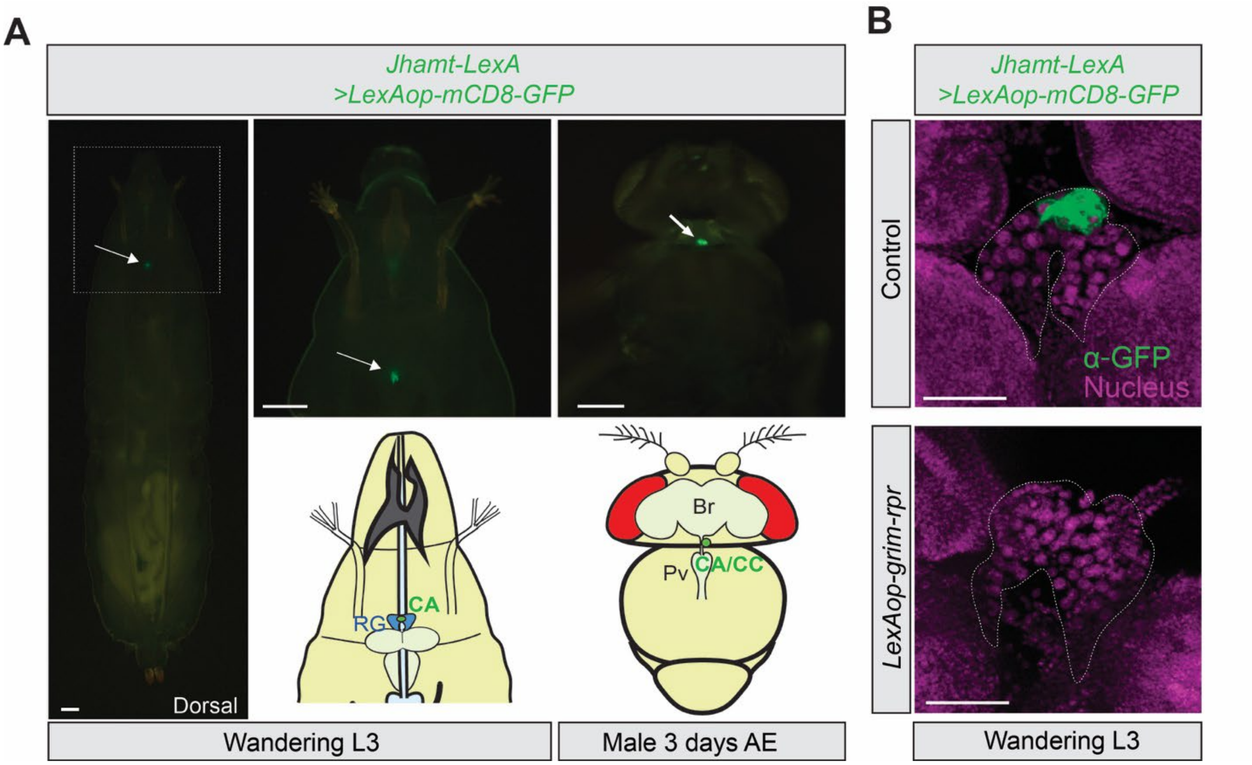

Figure S1. Visualization of *Jhamt-LexA* expression patterns.

(**A**) Expression patterns of *Jhamt-LexA* visualized by *LexAop-mCD8::GFP* expression. The corpus allatum (CA) is indicated by arrows. *Jhamt-LexA* is specifically expressed in the CA of wandering third instar (L3) larvae and adults. In the image of a whole larva, the anterior region indicated by the square is enlarged. Scale bars, 200 µm. RG, ring gland. AE, after eclosion. Br, brain. Pv, proventriculus. CA/CC, corpus allatum/corpora cardiaca complex. (**B**) Apoptosis induction in the CA by CA-specific overexpression of *grim* and *reaper* (*rpr*). CA cells labeled with GFP were lost in CAX larvae. The ring gland is outlined with white dotted lines. GFP expression in the CA was detected by immunostaining. Scale bars, 50 µm.

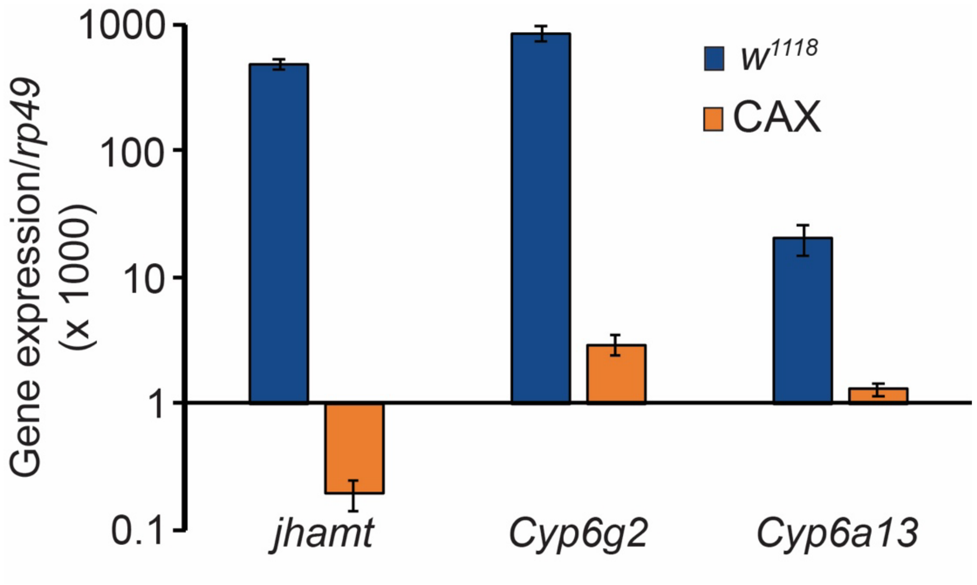

Figure S2. Absolute quantification of gene expression in the ring gland.

Absolute quantification of *jhamt*, *Cyp6g2*, and *Cyp6a13* expression in the ring gland of control (w^{1118}) and corpus allatum-ablated (CAX) larvae. Expression levels were normalized to a reference gene, *rp49*, in the same cDNA samples and shown on a log scale. A standard curve for each gene was generated using serial dilutions of a plasmid containing the target sequence. All target genes showed proportional amplification relative to the amount of the template plasmid (*jhamt*: efficiency = 95.7%, R^2 = 0.999; *Cyp6g2*: efficiency = 90.1%, R^2 = 0.995; *Cyp6a13*: efficiency = 119.5%, R^2 = 0.995; *rp49*: efficiency = 93.9%, R^2 = 0.999). Values are the means ± standard error (n = 3).

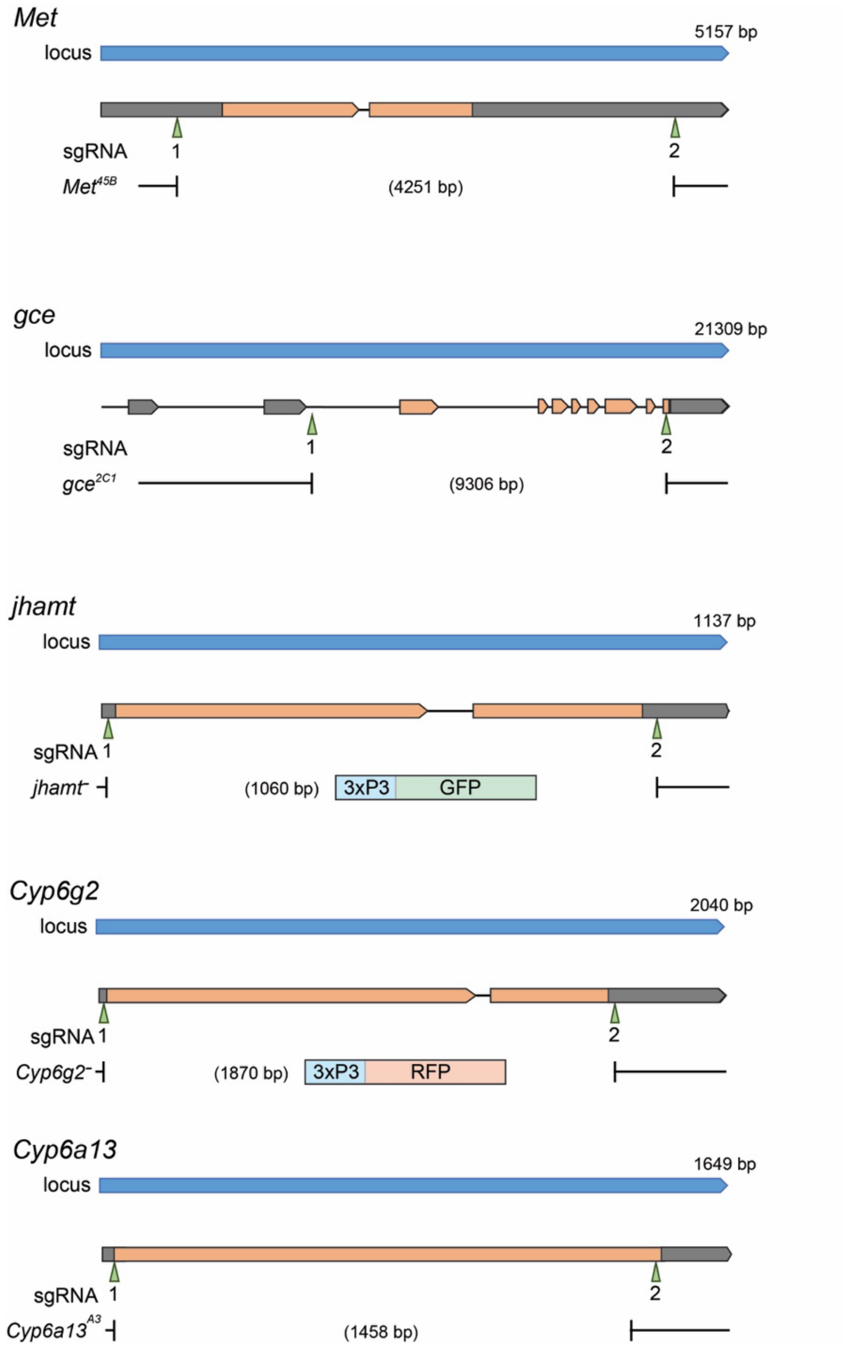

Figure S3. Mutagenesis of JH-related genes.

CRISPR-Cas9-mediated mutagenesis was performed using two single guide RNAs (sgRNAs) per target gene to delete most or all of the coding sequences (indicated in orange). A 3xP3-GFP sequence was inserted into the *jhamt* target site, and a 3xP3-RFP sequence was inserted into the *Cyp6g2* target site via homologous recombination.

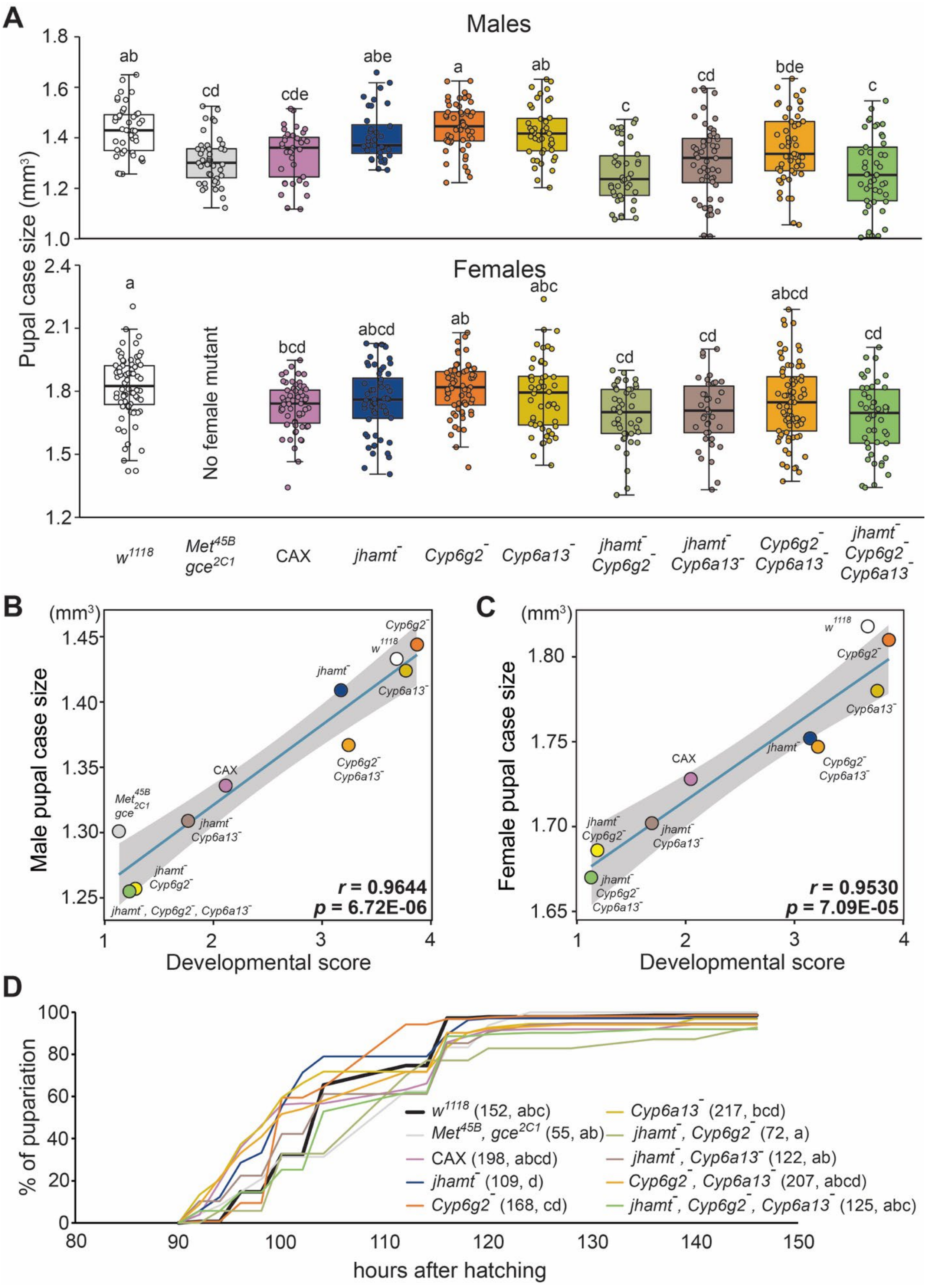

A
Males
Females
Pupal case size (mm³)
No female mutant
w1118
Met45B gce2C1
CAX
jhamt⁻
Cyp6g2⁻
Cyp6a13⁻
jhamt⁻ Cyp6g2⁻
jhamt⁻ Cyp6a13⁻
Cyp6g2⁻ Cyp6a13⁻
jhamt⁻ Cyp6g2⁻ Cyp6a13⁻
B
Male pupal case size
Developmental score
r = 0.9644
p = 6.72E-06
C
Female pupal case size
Developmental score
r = 0.9530
p = 7.09E-05
D
% of pupariation
hours after hatching
w1118 (152, abc)
Met45B, gce2C1 (55, ab)
CAX (198, abcd)
jhamt⁻ (109, d)
Cyp6g2⁻ (168, cd)
Cyp6a13⁻ (217, bcd)
jhamt⁻, Cyp6g2⁻ (72, a)
jhamt⁻, Cyp6a13⁻ (122, ab)
Cyp6g2⁻, Cyp6a13⁻ (207, abcd)
jhamt⁻, Cyp6g2⁻, Cyp6a13⁻ (125, abc)

Figure S4. Developmental phenotypes of CA-ablated flies and JH-related mutants.

(**A**) Pupal case size of CAX and the homozygous mutant males and females. Pupal case sizes of each genotype and sex are shown as scatter and box plots. Animals with severe lethal phenotypes tend to show smaller body sizes. *Met*/*gce* double mutant females were not available as these genes are on the X chromosome. $n = 36–73$. Different letters indicate statistically significant differences ($p < 0.05$) between groups (Tukey's honestly significant difference test). (**B, C**) Correlation between the developmental score and male (B) or female (C) body size. Developmental scores were calculated based on lethal stages. Strong positive correlations ($r > 0.95$, Pearson's correlation coefficient) were observed in both cases. (**D**) Pupariation timing after hatching was analyzed in CAX larvae and JH-related mutants. Most larvae entered the prepupal stage between 90 and 120 hours after hatching. Numbers in parentheses indicate the numbers of larvae analyzed. Different letters in parentheses indicate statistically significant differences between genotypes ($p < 0.05$, log-rank test).

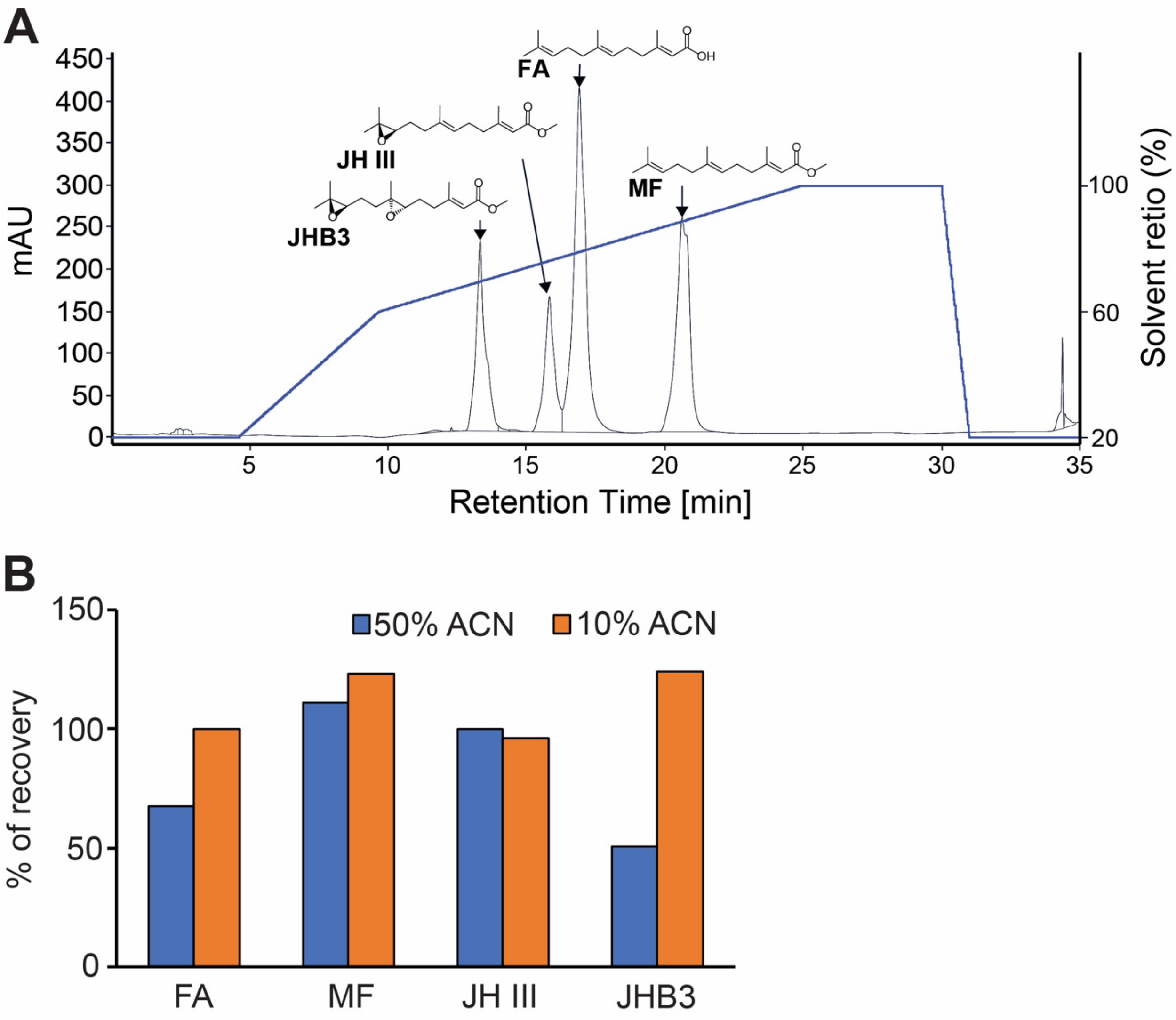

Figure S5. Simultaneous extraction of sesquiterpenoids.

(**A**) Chromatogram of the sesquiterpenoid mixture containing 1 µg each of authentic standards. (**B**) Recovery rate of each compound following hexane extraction from solutions containing different concentrations of acetonitrile (ACN). Note that recovery rates exceeding 100% are due to partial evaporation of methanol during concentration with the CentriVap concentrator. FA, farnesoic acid; MF, methyl farnesoate; JH III, juvenile hormone III; JHB3, juvenile hormone III bisepoxide.

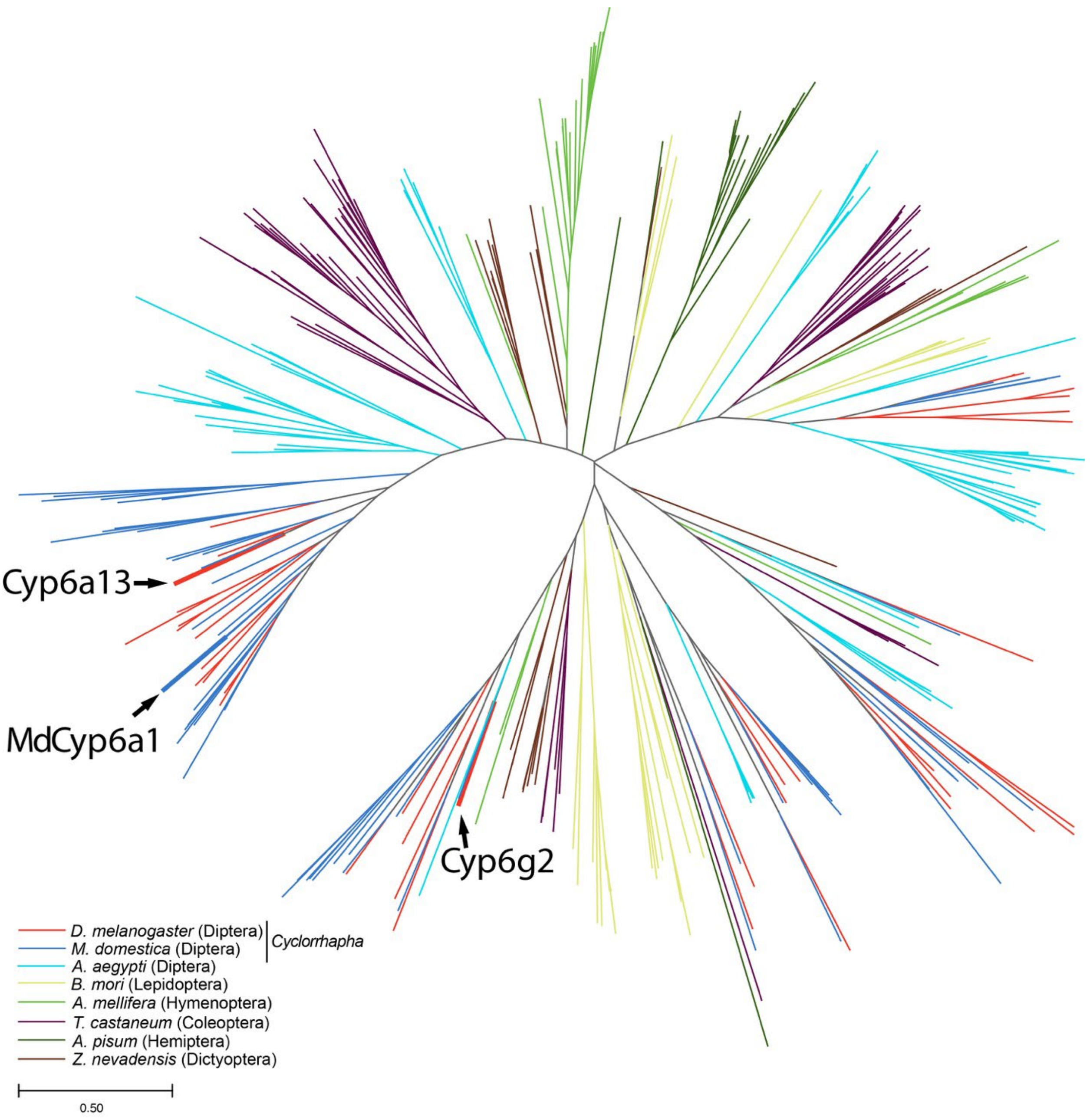

Figure S6. Phylogenetic analysis of enzymes in the insect CYP3 clan.

Unrooted maximum-likelihood phylogenetic tree of CYP3 clan enzymes in *Drosophila melanogaster*, *Musca domestica, Aedes aegypti, Bombyx mori*, *Apis mellifera*, *Tribolium castaneum*, *Acyrthosiphon pisum*, and *Zootermopsis nevadensis*. The scale bar indicates an evolutionary distance of 0.5 amino acid substitutions per site. Accession numbers of the enzymes analyzed are listed in Table S7.

Table S1. ***Drosophila*** **strains used in this study.**

Organisms: Strains	Source	Identifier
D. melanogaster: *w^{1118}*	Bloomington *Drosophila* Stock Center	BDSC: 5905
D. melanogaster: *jhamt-LexA*	Ohhara et al., 2018	
D. melanogaster: *LexAop-mCD8::GFP*	Bloomington *Drosophila* Stock Center	BDSC: 56173
D. melanogaster: *LexAop-grim, rpr*	Obtained from Tzumin Lee (University of Michigan)	N/A
D. melanogaster: *nos-Cas9*	National Institute of Genetics	CAS-0002
D. melanogaster: *U6-Cyp6a13 gRNA*	This paper	N/A
D. melanogaster: *jhamt-Gal4*	Obtained from Michael E. Adams (University of California, Riverside)	N/A
D. melanogaster: *Aug21-Gal4*	Obtained from Michael B. O'Connor (University of Minnesota)	N/A
D. melanogaster: *Met^{45B}*	This paper	N/A
D. melanogaster: *gce^{2C1}*	This paper	N/A
D. melanogaster: *$jhamt^{-}$*	This paper	N/A
D. melanogaster: *$Cyp6g2^{-}$*	This paper	N/A
D. melanogaster: *$Cyp6a13^{-}$*	This paper	N/A
D. melanogaster: *UAS-jhamt RNAi*	Vienna *Drosophila* Resource Center	VDRC: 103958
D. melanogaster: *UAS-Cyp6g2 RNAi*	Vienna *Drosophila* Resource Center	VDRC: 8131
D. melanogaster: *UAS-Cyp6a13 RNAi*	Vienna *Drosophila* Resource Center	VDRC: 4019
D. melanogaster: UAS-jhamt	This paper	N/A
D. melanogaster: UAS-Cyp6g2	This paper	N/A
D. melanogaster: UAS-Cyp6a13	This paper	N/A

Table S2. Plasmid constructs used in this study.

Recombinant DNA	Source	Identifier
cDNA AT13581 (*jhamt*)	*Drosophila* Genomics Resource Center	DGRC: 11840<br>FlyBase: FBcl0041099
cDNA IP03155 (*Cyp6g2*)	*Drosophila* Genomics Resource Center	DGRC: 1379871<br>FlyBase: FBcl0343443
cDNA LD25139 (*Cyp6a13*)	*Drosophila* Genomics Resource Center	DGRC: 2249<br>FlyBase: FBcl0175511
pcDNA-jhamt	This paper	N/A
pcDNA-Cyp6g2	This paper	N/A
pcDNA-Cyp6a13	This paper	N/A
pBFv-U6.2	National Institute of Genetics	N/A
pBFv-U6.2B	National Institute of Genetics	N/A
pUAST-jhamt	This paper	N/A
pUAST-Cyp6g2	This paper	N/A
pUAST-Cyp6a13	This paper	N/A

Table S3. Primers and oligonucleotides used in this study.

**Primers for qRT-PCR**			
Gene Name	CG Number	Forward (5'-3')	Reverse (5'-3')
jhamt	CG17330	TTTCTTGAGCGAATGCCTGC	AGGAGTCTTGCGAGCATAGGC
Cyp6g2	CG8859	AGGATACGGACGTACAGCAG	GCATGAACTCCAGTGACTCG
Cyp6a13	CG2397	ATGGAATTGGGTGAGGAGGG	AGAAGGACATGGTGGTGGAG
Cyp6a17	CG10241	ATTTCCCAAGTTATCGCGCC	CGCTTTTCCTTCGTCCGTAG
Cyp6v1	CG1829	TATCCCTCCACGATTCAGCC	TCAGGATCGTCACTATCGCC
Cyp9c1	CG3616	TGGGGCGGACTCAAAGTAAT	TCGATACTTCATGGCACCGA
Cyp310a1	CG10391	TATTGAACGTGTTTGCCGCA	TCGGTGCATAGCCAGTAGAG
Cyp4g1	CG3972	CGTCCAGACATCTACCCCAA	GTCCAGCGCTAAAGGGAATG
Cyp12e1	CG14680	CCACGTAACGCCAAGCTTTA	GTCCACGGCAAAGTCATCTG
phm	CG6578	CTTCTGGTCCAATCCCGACT	ATTCGTTTCTCTTTCCGCGG
Kr-h1	CG45974	GAATACGACATAACAGCC	CGATTTCCGTGAATATGTTCT
rp49	CG7939	AGCTGTCGCACAAATGGCGCAAGC	TTGAATCCGGTGGGCAGCATGTGG
**Oligonucleotides for generating gRNAs**			
Allele	Target ID	Forward (5'-3')	Reverse (5'-3')
Met^{45B}	1	CTTCGAAGGAGAGTGAGCGAGAGA	AAACTCTCTCGCTCACTCTCCTTC
	2	CTTCGTTGGTGATGGGCGCAGAGA	AAACTCTCTGCGCCCATCACCAAC
gce^{2C1}	1	CTTCGACTGAATCTGTCGGTGCAA	AAACTTGCACCGACAGATTCAGTC
	2	CTTCGTGATGGAGAGCGCCCGATC	AAACGATCGGGCGCTCTCCATCAC
Cyp6a13^{-}	1	CTTCGAGTACCAGGAGCGTCAGCA	AAACTGCTGACGCTCCTGGTACTC
	2	CTTCGGAGGGAGTTCATTTGAGGA	AAACTCCTCAAATGAACTCCCTCC
**Primers for screening CRISPR mutants**			
Allele		Forward (5'-3')	Reverse (5'-3')
Met^{45B}		GCCACCAGCAACAGCAACAACAAT	CGCTGGGGAATTAGCATTGGACTT
gce^{2C1}		GTAGCAGCCCAAGGACTATACCAT	CCATCAGTTGACCGAACGAGAAGT
Cyp6a13-		TGTGTCAGCGCTTCAAGTTC	ACATATTTCGCGCTGTCAGG
**Primers for generating *in situ* hybridization probes**			
Gene Name		Sequence (5'-3')	
jhamt	Forward	GTAATACGACTCACTATAGGGCACTCCTGGATGTGGGTTCAG	
	Reverse	GTGTATTTAGGTGACACTATAGGCATAGGCCACCACCAATTT	
Cyp6g2	Forward	GTAATACGACTCACTATAGGGCGTGCCAACAGTTTCACGGAT	
	Reverse	GTGTATTTAGGTGACACTATAGCCACCTTCGCCTGGAGATAT	
Cyp6a13	Forward	GTAATACGACTCACTATAGGGCACGGATATCAACCTGCGGAT	
	Reverse	GTGTATTTAGGTGACACTATAGAGAAGGACATGGTGGTGGAG	

Table S5. MS Settings to quantify each compound on the GC-MS.

Compound	Retention time (min)	Ion Polarity	Window (min)	Parent Mass	Product Mass	Collision Energy (V)
Citronellol	5.87	Positive	0.2	81.0	79.1	10
Methylfarnesoate	10.12	Positive	0.2	114.1	83.1	10
Juvenile hormone III	11.01	Positive	0.2	85.1	59.1	10

Table S6. MS Settings to quantify each compound on the LC-MS.

Compound	Retention time (min)	Ion Polarity	Window (min)	Parent Mass	Product Mass	Collision Energy (V)
Farnesoic acid	14.23	Negative	1.0	235.2	98.2	12
Juvenile hormone III	13.44	Positive	1.1	267.2	235.0	12
Juvenile hormone III-d3	13.41	Positive	0.9	270.2	153.2	20
Juvenile hormone bisepoxide	10.79	Positive	1.25	283.2	233.0	18
